# Knowledge of oxygen therapy among healthcare professionals in non-intubated patients: a cross-sectional study in Somalia

**DOI:** 10.1186/s12909-026-09766-8

**Published:** 2026-06-26

**Authors:** Pinar Duman Aydin, Gamze Küçükosman, Abdullahi Osman Mohamed, Dilek Konukbay, Adile Maşalacı, Onur Mutlu

**Affiliations:** 1 Department of Anesthesiology and Reanimation, Trabzon Kanuni Training and Research Hospital, Trabzon, Türkiye; 2 Department of Anesthesiology and Reanimation, Trabzon Kanuni Training and Research Hospital, University of Health Sciences, Trabzon, Türkiye; 3 Department of Anesthesiology and Reanimation, Mogadishu Recep Tayyip Erdoğan Training and Research Hospital, Mogadishu, Somalia; 4 Department of Pediatric Nursing, Gülhane Faculty of Nursing, University of Health Sciences, Ankara, Türkiye; 5 Faculty of Health Sciences, Mogadishu Recep Tayyip Erdoğan Training and Research Hospital, Mogadishu, Somalia; 6 Department of Anesthesiology and Reanimation, Faculty of Medicine, Trabzon University, Trabzon, Türkiye; 7 Department of Computer Science, Faculty of Science, Karadeniz Technical University, Trabzon, Türkiye

**Keywords:** Healthcare professionals, Knowledge level, Oxygen therapy, Somalia, Training

## Abstract

**Background:**

Although oxygen therapy (OT) is a fundamental and life-saving intervention in the management of hypoxemia, it may lead to serious complications when applied incorrectly or in an uncontrolled manner. The aim of this study is to evaluate the knowledge levels and attitudes of healthcare professionals working in a tertiary hospital in Somalia regarding OT administered to non-intubated patients.

**Methods:**

This descriptive cross-sectional study was conducted between 17 February and 2 March 2025 at Mogadishu Recep Tayyip Erdoğan Training and Research Hospital. A structured 23-item survey evaluating knowledge and attitudes related to OT was administered face-to-face to healthcare professionals consisting of nurses, resident physicians, and attending physicians. Knowledge levels were classified as poor (< 60%), moderate (60–79%), and good (≥ 80%) based on the percentage of correct responses. *p* < 0.05 was considered statistically significant.

**Results:**

A total of 195 healthcare professionals participated in the study (42.6% nurses, 35.9% resident physicians, and 21.5% attending physicians). Overall, 49.2% of the participants (95% CI: 42.2% – 56.2%) reported knowing how to administer OT, though knowledge levels were predominantly poor across all professional groups regarding specific technical parameters, and 70% of nurses and 90% of physicians stated that OT training should be received (*p* = 0.223). The rate of receiving training before starting duty was higher among nurses (71.1%) than among residents (44.3%) (*p* = 0.003). As training sources, nurses more frequently reported school and orientation training, while attending physicians more frequently reported conferences/course programs (*p* < 0.001). Guideline use rates were similar across groups and were generally limited. Knowledge levels regarding low- and high-flow OT systems, indications, and monitoring parameters were poor in all groups. Knowledge of oxygen toxicity was higher among physicians than nurses (*p* = 0.001), and no significant difference was found among the groups in terms of awareness of morbidity and mortality (*p* = 0.189).

**Conclusions:**

This study provides descriptive and hypothesis-generating data highlighting significant knowledge gaps regarding OT among healthcare professionals in a resource-limited setting. The findings suggest a critical need for standardized protocols and continuous training programs, though future prospective studies are required to determine the causal relationships between these knowledge gaps and actual clinical practice or patient outcomes.

**Supplementary Information:**

The online version contains supplementary material available at https://doi.org/10.1186/s12909-026-09766-8.

## Background

Oxygen (O₂) is an essential molecule required for sustaining aerobic metabolism and is present in the atmosphere at approximately 21%. Because of its central role in correcting tissue hypoxia, oxygen therapy (OT) remains one of the most frequently administered treatments in acute and critical care settings. A decrease in arterial O_2_ concentration may lead to impaired cellular energy production, organ dysfunction, and, if left untreated, death [1].

Oxygen therapy aims to increase tissue oxygenation by delivering O_2_ at a concentration higher than that of ambient air and is one of the most frequently performed supportive treatments in acute care practices [2]. International guidelines recommend that O_2_ be considered a pharmacological agent and administered through titration according to specific target saturation ranges. The World Health Organization classifies O_2_ among essential medicines and emphasizes its life-saving role, particularly in conditions such as acute respiratory failure, sepsis, shock, and severe anemia [3, 4]. However, in recent years, evidence regarding the potential harms of liberal O_2_ use has increased. Studies reporting hyperoxia-related oxidative stress, absorption atelectasis, vasoconstriction, pulmonary injury, and increased mortality risk have led to a re-evaluation of the safe limits of O_2_ use [4, 5]. Particularly in non-intubated patients, the appropriate use of different systems such as nasal cannulas, simple face masks, venturi masks, and high-flow nasal cannulas with proper indications and flow values requires knowledge and experience [5]. The clinical imperative for precise O_2_ titration has been unequivocally established by recent landmark clinical trials that prompted a paradigm shift toward conservative OT [6–8]. The Oxygen- Intensive Care Unit (ICU) trial demonstrated a significant mortality benefit associated with conservative O_2_ strategies in critically ill patients [6]. Subsequently, the ICU-ROX (Intensive Care Unit Randomized Trial Comparing Two Approaches to Oxygen Therapy) trial and the LOCO_2_ (Liberal Oxygenation versus Conservative Oxygenation in Acute Respiratory Distress Syndrome) trial further investigated conservative O_2_ targets in mechanically ventilated adults and acute respiratory distress syndrome, respectively. Together, these trials underscore the high clinical stakes of OT, proving that knowledge deficits in O_2_ prescribing do not merely represent theoretical gaps, but carry profound implications for patient survival and the prevention of iatrogenic harm. Together, these studies underscore the clinical importance of appropriate OT and demonstrate that deficiencies in O_2_ prescribing and administration may have important consequences for patient safety and outcomes [7, 8]. However, it was reported that due to fear of hypoxia, O_2_ is often administered in an uncontrolled manner or at unnecessarily high doses in clinical practice [9].

Although the prescription of OT is the responsibility of physicians, the maintenance of its administration, patient monitoring, and early detection of complications are duties that are largely carried out by nurses [1]. Therefore, the knowledge levels of healthcare professionals regarding OT are critically important for patient safety. In the literature, especially in developing countries, it has been reported that healthcare workers have knowledge deficiencies regarding OT, limited adherence to guidelines, and a lack of standardized protocols. These deficiencies have been associated with inappropriate O₂ administration practices, including unnecessary O₂ use, failure to achieve recommended target saturation ranges, and inconsistent monitoring of patients receiving OT [10–12]. However, data evaluating healthcare professionals’ knowledge and practices regarding OT in Africa remain limited, and evidence from Somalia is particularly scarce. Furthermore, recent reports from Somalia have highlighted important gaps in healthcare workers’ training on OT and hypoxemia management [13].

The gap in the current literature suggests that OT in low- and middle-income countries is performed mostly based on experience rather than knowledge. To ensure the safe administration of OT in non-intubated patients, it is necessary to determine the knowledge levels of healthcare professionals and identify potential deficiencies.

This study aims to evaluate the knowledge levels and attitudes of nurses and physicians working in a tertiary hospital in Somalia regarding OT administered to non-intubated patients and analyze current practices in light of international guidelines.

## Methods

### Study design and ethical approval

The descriptive and cross-sectional study was conducted between 17 February and 2 March 2025 after obtaining approval from the Ethics Committee Mogadishu Recep Tayyip Erdoğan Training and Research Hospital (Decision No: MSTH/21116) and written informed consent from the participants. The study was conducted in accordance with the principles of the Declaration of Helsinki. This study was reported in accordance with the Strengthening the Reporting of Observational Studies in Epidemiology (STROBE) statement for cross-sectional studies.

### Study population

The study population consisted of all physicians and nurses working in the hospital. It was aimed to include all healthcare professionals who could be reached during the study period and voluntarily agreed to participate. However, since participation was voluntary, the final sample was not considered a true census of the hospital workforce but was formed using a convenience sampling approach. Healthcare professionals who refused to participate, had previously completed the questionnaire, were on leave or sick leave during the study period, or submitted incomplete questionnaires were excluded from the study. To ensure transparency, the distribution of non-participants (those who refused participation, were on leave, or submitted incomplete questionnaires) was documented. A total of 99 incomplete questionnaires were excluded from the analyses because they contained substantial missing data (> 50%). This approach was adopted to preserve the integrity of the final dataset.

### Study variables

Primary outcome variable: overall knowledge level regarding OT (poor/moderate/good).

Secondary outcome variables:

- Awareness of O_2_ toxicity
- Awareness of the relationship between morbidity/mortality and O_2_ administration
- Protocol/guideline use status
- Training status

Independent variables: profession (nurse, resident physician, attending physician), age, gender, years of professional experience, and unit of employment.

### Data collection tool

The data collection instrument was a structured survey form developed by the researchers based on a literature review and previously published studies on OT knowledge [12, 14]. The questionnaire was not a previously validated instrument. To assess content validity and feasibility, the draft form was pilot tested with 10 healthcare professionals working in the same hospital who were not included in the main study sample. Necessary revisions were made based on participant feedback, and the final version of the survey form was created.

The survey form consisted of 23 questions. The first section included 6 questions evaluating the sociodemographic and professional characteristics of the participants (age, gender, profession, years of professional experience, unit of employment, and duration of work in the unit). The second section included 17 questions measuring training status and knowledge levels regarding OT. Eight questions contained multiple correct response options, and participants were informed that they could select more than one answer. These eight questions yielded a total of 50 individual answer items. In addition, nine questions had a single correct response, resulting in a total of 59 evaluable knowledge items. The overall knowledge score for each participant was calculated by dividing the number of correctly answered items by 59 and multiplying by 100. The survey form is presented in Appendix 1.

The knowledge levels of the participants were classified as poor (< 60%), moderate (60–79%), and good (≥ 80%) based on the percentage of their correct responses. These classification thresholds were adopted from previously published OT knowledge studies to facilitate comparison with existing literature [10–14]. The reliability of the survey form was evaluated using Cronbach’s alpha coefficient, which was found to be 0.827. This value indicated good internal consistency according to both classical psychometric criteria and contemporary measurement-property reporting standards [15].

### Data collection process

Data were collected by administering the survey form face-to-face to physicians and nurses working in the emergency department, adult and pediatric clinics, and intensive care units of the hospital. The participants were asked to complete the form independently based on their own knowledge and experience. They were informed that responses were anonymous and would be used solely for scientific purposes. The data were collected and filed by an anesthesia resident who did not participate in the study and had no prior knowledge of the study. Upon completion of the study, the data were stored anonymously and were accessible only to the research team for analysis purposes.

### Bias and control measures

Since the study was based on the self-reports of the participants, there was a risk of information bias. To reduce this risk, the survey was administered anonymously, and care was taken to avoid influencing the participants. Due to the single-center design, selection bias and limitations in generalizability should be considered.

### Sample size

The required sample size was calculated using the G*Power (version 3.1) software for an a priori power analysis. Pearson’s chi-squared test of independence was accepted as the primary analysis method. Assuming a medium effect size (w = 0.30), a 5% significance level (α = 0.05), and 95% statistical power (1-β = 0.95), it was determined that at least 176 participants would be sufficient for three professional groups and a three-category outcome variable.

### Statistical analysis

All statistical analyses were performed using IBM SPSS Statistics for Windows, version 27.0 (IBM Corp., Armonk, NY, USA). Categorical variables are presented as frequency (n) and percentage (%) values, accompanied by 95% confidence intervals (CIs) to evaluate the precision of the estimates. Pearson’s chi-squared test was used for comparisons between groups, and the magnitude of these differences was quantified using Cramér’s V for effect size. Bonferroni-corrected post hoc analyses were performed to determine which groups accounted for significant differences, utilizing an adjusted significance threshold of *p* < 0.0167. Fisher’s exact test was used when the assumptions of the chi-squared test were not met. In all overall analyses, *p* < 0.05 was considered statistically significant.

## Results

**Fig. 1:**
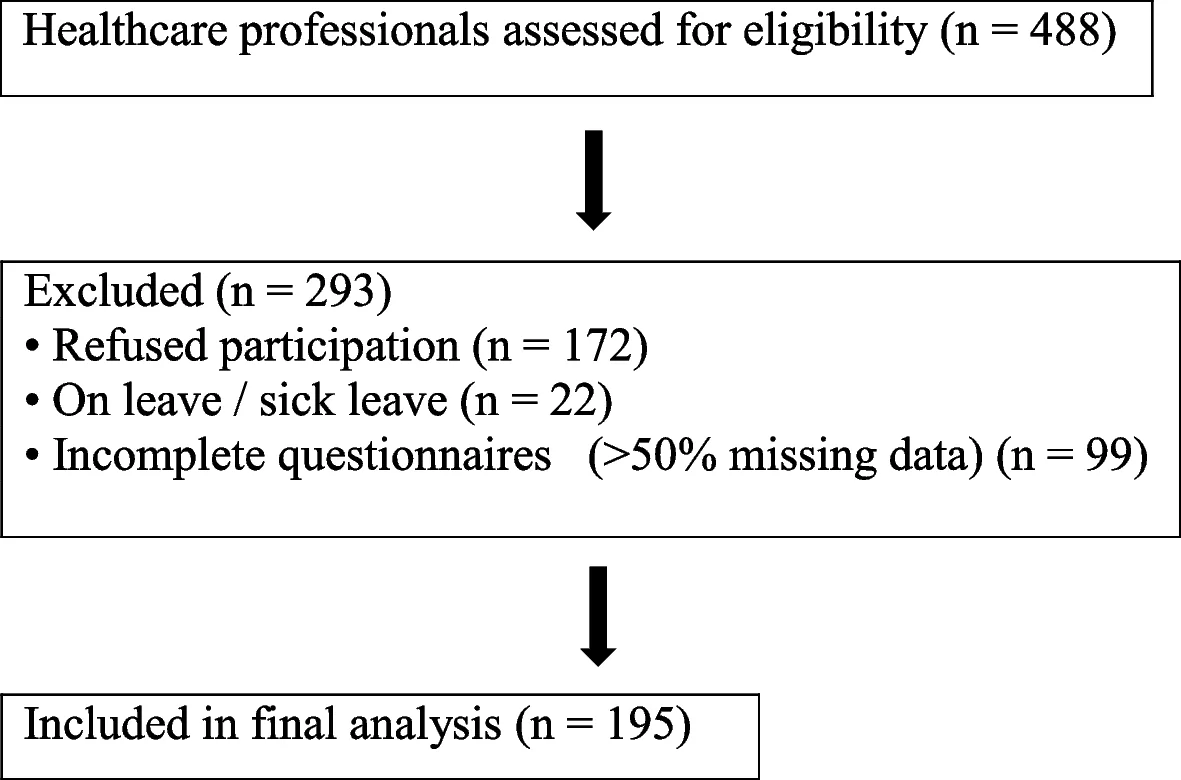
Flow diagram of participant recruitment, exclusions, and final sample selection

During the study period, 488 healthcare professionals (198 physicians and 290 nurses) were reached. The participation rate was 40%, and analyses were conducted on data collected from 195 participants. The participant recruitment and selection process is summarized in Fig. 1.

Among the participants, 42.6% were nurses (*n* = 83), 35.9% were resident physicians (*n* = 70), and 21.5% were attending physicians (*n* = 42). Demographic and professional characteristics, including age groups, gender distributions, years of professional experience, units of employment, and duration of work in respective units, are presented in Table 1.

**Table 1:** Demographic and professional characteristics of the participants

		**n**	**%**
Gender	Male	114	58.5
	Female	81	41.5
Age group (years)	< 20	13	6.7
	21–39	154	79.0
	40–59	28	14.3
Years of professional experience			
	< 6	95	48.7
	6–10	65	33.3
	11–15	21	10.8
	16–20	14	7.2
Unit of employment	Internal Medicine	84	43.1
	Surgical Departments	40	20.5
	Intensive Care Unit	49	25.1
	Emergency Department	22	11.3
Duration of work in the unit (years)	< 5	120	61.5
	6–10	52	26.7
	11–15	13	6.7
	16–20	8	4.1
	> 21	2	1.0

Comparison of knowledge, training status, and educational needs regarding OT among professional groups are presented in Table 2. Overall, 49.2% of the participants stated that they knew how to administer OT. A statistically significant relationship was found between professional groups and receiving OT training before starting duty. The post hoc analysis showed that this significant difference was between nurses and resident physicians. The rate of receiving training before starting duty was 71% (*n* = 59) among nurses and 44.3% (*n* = 31) among resident physicians (*p* = 0.003). No significant difference was found in the comparisons between attending physicians and other professional groups. The proportion of those who believed that training on OT is necessary was 70% among nurses and 90% among physicians (Table 2).

**Table 2:** Comparison of oxygen therapy knowledge, training status, and educational needs among professional groups

	**Nurse** ***n* = 83 (%)**	**Resident physicians** ***n* = 70 (%)**	**Attending physicians *n* = 42 (%)**	**p**
Knowledge of OT administered?				
Yes	45(54.2)	32(45.7)	19(45.2)	0.782*
No	19(22.9)	21(30)	13(31)	
Partially	19(22.9)	17(24.3)	10(23.8)	
Received OT training before starting work in current unit?				
Yes	59(71.1)**	31(44.3)**	23(54.8)	0.003*
No	24(28.9)**	39(55.7)**	19(45.2)	
Need for OT training?				
Definitely required	67(70.7)	63(90)	38(90.5)	0.223*
Definitely not required	4(4.8)	4(5.7)	3(7.1)	
No opinion	12(14.5)	3(4.3)	1(2.4)	

*OT *Oxygen therapy

^*^Pearson’s chi-square test

^**^Bonferroni-corrected post hoc test (significance threshold: *p* < 0.0167)

In the evaluations of the training sources of the participants who stated that they had received OT training [nurses (*n* = 59), residents (*n* = 31), and attending physicians (*n* = 23)], a significant difference was found among professional groups (*p* < 0.001). The post hoc analysis showed that the difference resulted particularly from the categories of school training, orientation training, and conferences/course programs. The rates of receiving school and orientation training were higher among nurses (*n* = 18, 30.5%; *n* = 8, 13.6%) compared to residents (*n* = 7, 22.6%; *n* = 1, 3.2%) and attending physicians (*n* = 2, 8.7%; *n* = 1, 4.3%) (*p* = 0.01). On the other hand, the rate of reporting conferences/course programs as a training source was significantly higher among attending physicians (*n* = 9, 39%) compared to other professional groups (*p* < 0.05). Although in-service training was the most frequently reported training source in all groups (44%, 48.4%, and 34.8%, respectively, for nurses, resident physicians, and attending physicians), no significant difference was found among the groups for this variable. No significant difference was found among professional groups regarding “other” training sources. The proportions of participants who reported following specific protocols or guidelines when administering OT were 54.2% among nurses, 44.3% among resident physicians, and 61.9% among attending physicians, with no statistically significant difference between the professional groups (*p* > 0.103).

Notably, in questions regarding the flow values required to achieve 100% Fraction of inspired oxygen (FiO_2_) via face masks and nasal cannulas, as well as the key parameters used for patient monitoring, 100% of the participants across all professional groups scored at the poor level. Because there was zero variance across these three domains, statistical between-group comparisons could not be performed, and they were excluded from the tabular presentation.

When evaluating the knowledge domains presented in Table 3, knowledge levels regarding low- and high-flow systems, acute and chronic OT indications, and the benefits of guideline adherence showed similar distributions across professional groups. Although no statistically significant differences were found in these domains, the effect sizes were generally small (Cramér's V ranging from 0.104 to 0.147). However, for the evaluation of clinical outcomes expected in patients receiving OT, a potentially noteworthy between-group difference was observed: 91.6% of nurses scored at the poor level, compared to 77.1% of resident physicians and 73.8% of attending physicians. Although this comparison did not reach conventional statistical significance (**χ**^**2**^ = 9.353, *p* = 0.053, Cramér's V = 0.155), the borderline p-value and the observed percentage gap suggest that the study may have been underpowered to detect a moderate but clinically meaningful difference in this specific domain. A similar non-significant trend was observed in knowledge regarding clinical outcomes when OT is inadequately administered (**χ**^**2**^ = 5.658, df = 4, *p* = 0.226, Cramér's V = 0.120) (Table 3).

**Table 3:** Knowledge levels regarding the clinical application of oxygen therapy according to professional groups

	**Nurse (*n* = 83) (%)**	**Resident physicians (*n* = 70) (%)**	**Attending physicians (*n* = 42) (%)**	**χ**^**2**^	**Cramér's V**	***p*-value**
Low-flow O₂ systems						
Poor level	72 (86.8)	63 (90)	33 (78.6)	4.20	0.104	0.380*
Moderate level	9 (10.8)	6 (8.6)	6 (14.3)			
Good level	2 (2.4)	1 (1.4)	3 (7.1)			
High-flow O₂ systems						
Poor level	72 (86.8)	63 (90)	32 (76.2)	6.46	0.129	0.168*
Moderate level	10 (12)	6 (8.6)	7 (16.7)			
Good level	1 (1.2)	1 (1.4)	3 (7.1)			
Acute OT indications						
Poor level	79 (95.2)	68 (97.1)	37 (88.1)	4.24	0.147	0.121*
Moderate level	4 (4.8)	2 (2.9)	5 (11.9)			
Chronic OT indications						
Poor level	82 (98.8)	70 (100)	40 (95.2)	4.08	0.145	0.133*
Moderate level	1 (1.2)	0 (0.0)	2 (4.8)			
Expected clinical outcomes of OT						
Poor level	76(91.6)**	54 (77.1)	31 (73.8)	9.353	0.155	0.053*
Moderate level	5(6.0)**	14 (20.0)	10 (23.8)			
Good level	2 (2.4)	2 (2.9)	1 (2.4)			
Clinical outcomes of inadequate OT						
Poor level	83 (100.0)	66 (94.3)	40 (96.4)	5.658	0.120	0.226*
Moderate level	0 (0.0)	3 (4.3)	2 (2.6)			
Good level	0 (0.0)	1 (1.4)	0 (0.0)			
Benefits of OT guideline adherence						
Poor level	74 (89.2)	61 (87.1)	32 (76.2)	4.013	0.143	0.134*
Moderate level	9 (10.8)	9 (12.9)	10 (23.8)			

*OT* Oxygen therapy, *FiO₂* Fraction of inspired oxygen

^***^Comparison between groups could not be performed

^*^Pearson’s chi-square test

^**^Bonferroni-corrected post hoc test

No statistically significant difference was found among professional groups in terms of awareness of the relationship between O_2_ administration practices and morbidity and mortality (*p* = 0.189). However, compared to other groups, the awareness rate was higher among attending physicians at 83.3%.

Knowledge levels regarding O_2_ toxicity differed significantly among professional groups (*p* = 0.001). The proportion of those who stated that they had knowledge on this issue was 84.3% among resident physicians and 88.1% among attending physicians, whereas it was 60.2% among nurses.

The awareness levels of the participants regarding O_2_ toxicity and the effects of O_2_ administration practices on morbidity and mortality are presented in Table 4.

**Table 4:** Awareness of oxygen toxicity and oxygen-related morbidity and mortality by professional groups

	**Nurse** ***n* = 83 (%)**	**Resident physicians *n* = 70 (%)**	**Attending physicians *n* = 42 (%)**		**p**
Awareness of oxygen-related morbidity and mortality					
Yes	52(62.7)	51(72.9)		35(83.3)	0.189
No	7(8.4)	5(7.1)		2(4.8)	
Insufficient knowledge	24(28.9)	14(20.0)		5(11.9)	
Awareness of oxygen toxicity					
Yes	50(60.2)**	59(84.3)**		37(88.1)**	0.001*
No	6(7.2)	3(4.3)		2(4.8)	
Insufficient knowledge	27(32.6)**	8(11.4)**		3(7.1)**	

^*^Pearson’s chi-square test

^**^Bonferroni-corrected post hoc test (adjusted significance threshold: *p* < 0.0167)

## Discussion

In this study, it was determined that the knowledge levels of healthcare professionals regarding OT administered to non-intubated patients were generally low. Although the participants largely acknowledged the clinical necessity of OT, it was observed that there were significant knowledge deficiencies in critical areas such as technical application steps, device selection, the flow–FiO₂ relationship, and monitoring parameters. It is noteworthy that awareness regarding O₂ toxicity was higher among physicians and more limited among nurses. Nevertheless, the low rate of protocol and guideline use suggested that standardization in clinical practice was insufficient.

Previous studies have demonstrated that healthcare professionals' knowledge regarding OT varies across settings, with many reporting deficiencies in appropriate device selection, determination of target oxygenation levels, monitoring parameters, and adherence to guidelines [1, 12, 14, 16–19]. In contrast, some studies have reported higher levels of knowledge and greater use of guidelines, which may be related to differences in healthcare system organization, continuing professional education, and the availability of local protocols [20–22]. Furthermore, a recent systematic review and meta-analysis by Aynalem et al. identified substantial deficiencies in OT-related knowledge, attitudes, and practices among healthcare professionals in multiple low- and middle-income countries [9]. Consistent with this body of evidence, our study revealed important knowledge gaps, particularly in technical application steps. These findings suggest that the deficiencies observed in the present study are not limited to a single institution but may reflect a broader challenge in OT education and implementation.

Previous studies have shown that knowledge regarding OT is largely acquired during undergraduate education, while opportunities for ongoing professional training remain limited [1, 14]. Similarly, a substantial proportion of healthcare professionals have been reported not to participate in OT-related educational activities after graduation, with most training being received during formal schooling [23, 24]. At the same time, studies demonstrating significant improvements in knowledge and clinical practice following structured educational interventions suggest that such programs can be effective [25]. Consistent with these findings, most participants in our study considered OT training necessary. Differences in educational exposure were also observed across professional groups; nurses more frequently reported receiving training before starting their duties, whereas attending physicians primarily acquired knowledge through conferences and professional courses. Nevertheless, the persistence of knowledge gaps despite previous training indicates that current educational activities may not provide sufficient continuity or reinforcement. The absence of regular refresher programs and standardized institutional protocols may further contribute to the maintenance of these deficiencies.

The appropriate administration of OT requires correct device selection, adjustment of flow rates, and continuous patient monitoring [14]. Despite this, previous studies have demonstrated a considerable gap between theoretical knowledge and clinical practice, indicating that healthcare professionals do not always translate their knowledge into evidence-based OT management [12, 26, 27]. Similar findings from anesthesia and intensive care settings suggest that oxygen use frequently deviates from international recommendations [28]. Standardized protocols have been associated with reductions in hospital length of stay, duration of oxygen therapy, and OT-related complications, highlighting the potential value of structured institutional approaches [29]. Although the present study did not assess the effects of protocol implementation on clinical practice or patient outcomes, our findings revealed limited awareness of guideline use across all professional groups. This observation is consistent with previous reports linking knowledge deficiencies in acute OT to poor familiarity with international guidelines and the absence of local protocols [16]. Together, these findings suggest that standardized OT practices have not yet been fully integrated into routine clinical care. In resource-limited healthcare settings such as Somalia, strengthening both educational initiatives and protocol implementation may help improve the quality and consistency of OT delivery.

Although OT is often regarded as a safe supportive intervention, inappropriate or excessive oxygen administration can result in clinically significant adverse effects. Hyperoxia-related absorption atelectasis, oxidative tissue injury, vasoconstriction, and respiratory suppression—particularly in patients with chronic hypercapnia—have been well documented in the literature. Consequently, oxygen should be prescribed and monitored in the same manner as other therapeutic agents, with clear indications, target saturation ranges, and titration strategies [3, 5]. Previous studies have reported limited awareness among healthcare professionals regarding the risks associated with oxygen therapy [1, 16]. In the present study, awareness of O₂ toxicity was higher among resident and attending physicians than among nurses, possibly reflecting differences in clinical responsibilities and educational exposure. Given that nurses are primarily responsible for the administration and monitoring of OT, this finding has important implications for patient safety. These results highlight the need for educational programs that emphasize oxygen-related adverse effects, target saturation ranges, and appropriate titration practices.

## Limitations

This study has several limitations. Although it represents, to our knowledge, the first empirical assessment of healthcare professionals’ knowledge regarding OT in Somalia and provides important baseline data for future research, its findings should be interpreted with caution. The cross-sectional, single-center design precludes causal inferences regarding the relationships between training, knowledge, and clinical practice, and limits the external validity of the results.

Although the professional distribution of participants reflected the overall hospital workforce, voluntary participation may have introduced selection bias, and non-response bias cannot be excluded because detailed information on non-participating staff was unavailable. In addition, the use of self-reported data may have resulted in response and social desirability bias.

The study did not include direct observation of clinical practice or chart audits; therefore, reported knowledge levels may not accurately reflect actual clinical behavior or patient outcomes. Furthermore, differences in professional responsibilities and exposure to OT administration across occupational groups may have influenced the observed findings.

Several questionnaire domains also demonstrated floor effects, with nearly all participants classified in the poor knowledge category. This may have reduced the discriminatory capacity of these items and limited the ability to detect meaningful differences between professional groups.

Finally, the study was conducted in a tertiary referral hospital with distinctive institutional characteristics, including international academic and healthcare collaborations. Consequently, the findings may not be representative of other healthcare facilities in Somalia. Larger multicenter studies involving diverse healthcare settings and professional groups are needed to confirm and extend these findings.

## Conclusion

The findings of this study indicate that healthcare professionals working in Somalia have important knowledge gaps regarding OT in non-intubated patients, particularly in technical application, monitoring parameters, and O_2_ toxicity. These findings suggest the potential value of structured educational programs and wider implementation of evidence-based OT protocols and guidelines. However, whether such interventions translate into measurable improvements in clinical practice and patient outcomes requires further investigation.

## Supplementary Information

Supplementary Material 1.

Supplementary Material 2.

Supplementary Material 3.

## Data Availability

The data that support the findings of this study are available from the corresponding author upon reasonable request.

## Abbreviations

ICU: Intensive Care Unit
ICU-ROX: Intensive Care Unit Randomized Trial Comparing Two Approaches to Oxygen Therapy
OT: Oxygen therapy
O_2_: Oxygen
FiO_2_: Fraction of inspired oxygen
