# Supplementary material for: Knowledge of oxygen therapy among healthcare professionals in non-intubated patients: a cross-sectional study in Somalia
Source: BMC Med Educ. 2026 Jun 26;26:1464. doi: 10.1186/s12909-026-09766-8 (PMC13573470; doi:10.1186/s12909-026-09766-8)
Supplement: Supplementary file 1 [file 12909_2026_9766_MOESM2_ESM.pdf]

Knowledge of Oxygen Therapy Among Healthcare Professionals in Non-Intubated Patients: A Cross-Sectional Study in Somalia

Dear Employee,

The following questions have been prepared to evaluate the attitudes and behaviors of healthcare professionals (doctors, nurses) working in our hospital about oxygen therapy. Volunteering is essential in answering the survey questions. You are not asked to write your name and surname on the forms given to you. In addition, your answers to the questions will be evaluated collectively, not individually, and confidentiality will be ensured by the researchers. After reading the statements, please mark the option that suits you with an X. The survey duration is approximately 15 minutes. If you have already responded to our survey, please do not do so again. Thank you for taking the time to participate in our survey.

QUESTIONS

1. **Your Age?**
   A. <20 year
   B. 21-39 year
   C. 40-59 year
   D. >60 year
2. **Your gender?**
   A. Male B. Female
3. **Your duty?**
   A. Nurse B. Resident physician C. Attending physician
4. **Number of years in your profession? (Including your training period)**
   A. 6-10 years
   B. 11-15 years
   C. 16-20 Years
5. **Which service and/ or intensive care unit (ICU) do you work in?**
   A. Internal Medicine
   B. Surgical Departments
   C. Intensive Care Unit (ICU)
   D. Emergency Department
6. **Your current service and/ or ICU tenure?**
   A. 0-5 years
   B. 6-10 years
   C. 11-15 years
   D. 16-20 Years
   E. 21 years and above
7. **Do you know how oxygen therapy is performed?**
   A. Yes B. Partially C. No
8. **Have you received any training on OT practices before taking up a position in the unit where you work?**
   A. Yes-I did (Go to question 9) B. No-I did not (Go to question 10)
9. **If you have received any training on OT practices, where have you received the training?**
   A. In-service training
   B. School education

C. Orientation training
D. Conference/course programs
E. Other, specify:

10. What do you think about being trained regarding OT practices?

A. Definitely necessary B. Definitely not necessary C. I don't have much idea about it

11. Do you use specific protocols or guidelines for OT practices?

A. Yes B. No

12. What are the ways to apply low flow system O_2 therapy? (You can mark multiple options?

A. Nasal cannula
B. Simple oxygen mask
C. Partial rebreather mask
D. Non-rebreather mask
E. I don't have enough knowledge yet

13. What are the ways to apply high flow system O_2 therapy? (You can mark multiple options)?

A. Venturi mask
B. Aerosol mask
C. High frequency O_2 (HFO)
D. Continuous positive airway pressure (CPAP)
E. I don't have enough knowledge yet

14. What is the L/min flow rate you apply which makes FiO_2: 100% equivalent while you are performing O_2therapy on a patient with a face mask?

A. 2-3 L/min B. 4-5 L/min C. 7-8 L/min D. None

15. What is the L/min flow rate you apply which makes FiO_2 : 100% equivalent while you are performing O_2therapy with nasal cannula on a patient?

A. 2-3 L/min B. 4-5 L/min C. 7-8 L/min D. None

16. What are the indications for short-term (acute) OT? (You can mark multiple options)?

A. Acute hypoxemia (PaO_2<60 mmHg, SaO_2<90%)
B. Cardiac and respiratory arrest
C. Hypotension (systolic blood pressure<100 mmHg)
D. Low cardiac output and metabolic acidosis
E. Respiratory distress (respiratory rate>24/min)
F. I don't have enough knowledge yet

17. Which of the following statements is true regarding long-term (chronic) OT? (You can mark multiple options)?

A. Fixes polycythemia.
B. Reduces pulmonary artery pressure.
C. Fixes right and left ventricular functions.
D. It reduces severe desaturation periods in hypoxemic patients with COPD.
E. Reduces dyspnea in hypoxemic patients with COPD.
F. Fixes neuropsychiatric functions.
G. Increases exercise tolerance.
H. Reduces the need for hospitalization.
İ. I don't have enough knowledge yet

18. Which of the following clinical outcomes would you expect to observe in patients receiving oxygen support (You may select multiple options)?

A. Reduction in infections
B. Decrease in complications
C. Acceleration of wound healing
D. Reduced length of hospital stay
E. I don't have enough knowledge yet

19. Which of the following clinical outcomes would you expect to observe in cases where you feel that oxygen support is not adequately provided (You may select multiple options)?

A. Increased risk of infection
B. Delay in wound healing
C. Complications in surgical incisions, sutures and anastomoses
D. Muscle weakness
E. Cardiac dysfunctions
F. Metabolic acidosis
G. Respiratory dysfunctions
H. Difficulties in switching to spontaneous breathing in ventilator-supported patients
İ. Longer recovery and hospital stay
J. I don't have enough knowledge yet

20. Which of the following parameters do you use the most frequently in the follow-up of the patient receiving oxygen support (You can select multiple options)?

A. Peripheral SpO_2
B. PaO_2in arterial blood gas
C. $PaCO_2$ in arterial blood gas
D. SpO_2in arterial blood gas
E. Hemoglobin
F. I don't have enough knowledge yet

21. Are you aware of the relationship betweenO_2 applications and morbidity and mortality?

A. Yes B. No C. I don't have much idea about it

22. Which of the following do you think is/are among contribution/s brought by following the guidelines regarding OT in patients hospitalized in intensive care units? (You can mark multiple options)?

A. I think it will reduce the length of stay of patients in intensive care units.
B. I think it will contribute to the economy.
C. I think that complications such as infections and pressure sores could occur less frequently by following the precautions.
D. I don't think it will contribute to anything.

23. Are you knowledgeable of oxygen toxicity?

A. Yes
B. No
C. I don't have enough knowledge yet

Thank you for your participation.
