# Supplementary material for: Knowledge of oxygen therapy among healthcare professionals in non-intubated patients: a cross-sectional study in Somalia
Source: BMC Med Educ. 2026 Jun 26;26:1464. doi: 10.1186/s12909-026-09766-8 (PMC13573470; doi:10.1186/s12909-026-09766-8)
Supplement: Supplementary file 2 [file 12909_2026_9766_MOESM3_ESM.pdf]

STROBE Statement—Checklist of items that should be included in reports of cross-sectional studies

Item No	Recommendation	Reported on Page No
1(a)	Indicate the study's design with a commonly used term in the title or abstract	Title, Abstract (pp. 1–2)
1(b)	Provide an informative and balanced summary in the abstract	Abstract (pp. 2–3)
2	Explain the scientific background and rationale	Background (pp. 3–5)
3	State specific objectives and hypotheses	Background, final paragraph (p. 5)
4	Present key elements of study design early in the paper	Methods – Study design (p. 6)
5	Describe setting, locations, and relevant dates	Methods – Study design (p. 6)
6(a)	Give eligibility criteria and sources/methods of participant selection	Methods – Study population (pp. 6–7)
7	Clearly define outcomes, exposures, predictors, confounders	Methods – Study variables (p. 6)
8*	Give sources of data and details of assessment methods	Methods – Data collection tool (pp. 7–8)
9	Describe efforts to address potential sources of bias	Methods – Bias and control measures (p. 8)
10	Explain how study size was arrived at	Methods – Sample size (p. 9)
11	Explain handling of quantitative variables	Methods – Data collection tool (pp. 7–8)
12(a)	Describe all statistical methods	Statistical Analysis (p. 9)
12(b)	Describe methods used to examine subgroups and interactions	Statistical Analysis (p. 9)
12(c)	Explain how missing data were addressed	Study population (p. 6), Results (p. 10)
12(d)	If applicable, describe analytical methods taking account of sampling strategy	Not applicable – convenience sample without weighting
12(e)	Describe sensitivity analyses	No sensitivity analyses were performed
13(a)	Report numbers of individuals at each stage of study	Results, Figure 1 (pp. 9–10)
13(b)	Give reasons for non-participation at each stage	Results, Figure 1 (p. 10)
13(c)	Consider use of a flow diagram	Figure 1 (p. 10)
14(a)	Give characteristics of study participants	Table 1 (pp. 21–22)
14(b)	Indicate number of participants with missing data	Results (p. 10)
15*	Report numbers of outcome events or summary measures	Tables 2–4 (pp. 22–26)

Item No	Recommendation	Reported on Page No
16(a)	Give unadjusted estimates and precision measures	Tables 2–4, Results (pp. 10–12)
16(b)	Report category boundaries when continuous variables categorized	Methods – Data collection tool (p. 8)
16(c)	If relevant, translate relative risk into absolute risk	Not applicable – no risk estimates calculated
17	Report other analyses done	Results (pp. 10–12)
18	Summarize key results with reference to objectives	Discussion, first paragraph (p. 12)
19	Discuss limitations and potential bias	Limitations (pp. 15–16)
20	Give cautious overall interpretation	Discussion and Conclusion (pp. 12–16)
21	Discuss generalizability of results	Limitations (p. 16)
22	Give source of funding and role of funders	Funding statement (p. 17)
